# Associations between functional autoantibodies targeting GPCRs, antinuclear antibodies, and inflammatory cytokines TNF-α: a cross-sectional study of 19,810 individuals

**DOI:** 10.3389/fimmu.2025.1743537

**Published:** 2026-01-19

**Authors:** Xin Chen, Brit Kieselbach, Bernhard K. Krämer, Volker von Baehr, Christoph Reichetzeder, Berthold Hocher

**Affiliations:** 1 Institute for Clinical Research and Systems Medicine, Health and Medical University, Potsdam, Germany; 2 Fifth Department of Medicine (Nephrology/Endocrinology/Rheumatology/Pneumology), University Medical Centre Mannheim, University of Heidelberg, Mannheim, Germany; 3 Institute of Medical Diagnostics (IMD), Berlin, Germany; 4 European Center for Angioscience, Medical Faculty Mannheim, University of Heidelberg, Heidelberg, Germany; 5 Reproductive and Genetic Hospital of CITIC-Xiangya, Changsha, China; 6 Institute of Reproductive and Stem Cell Engineering, NHC Key Laboratory of Human Stem Cell and Reproductive Engineering, School of Basic Medical Science, Central South University, Changsha, China

**Keywords:** antinuclear antibodies, autoantibodies, G protein-coupled receptors, sex, tumor necrosis factor alpha

## Abstract

**Introduction:**

Functional autoantibodies targeting G protein–coupled receptors (GPCR-AAbs) have increasingly been implicated in autoimmune and inflammatory diseases. However, their relationships with established autoimmune biomarkers, such as antinuclear antibodies (ANA), and key inflammatory cytokines remain insufficiently understood. This study aimed to investigate the associations between different functional GPCR-AAbs, ANA positivity, and inflammatory cytokines, with a particular focus on potential sex-specific effects.

**Methods:**

We conducted a cross-sectional analysis of 19,810 individuals from a large clinic-based cohort. Serum concentrations of functional GPCR-AAbs (Igβ1AR-AAb, Igβ2AR-AAb, IgM3MR-AAb, IgM4MR-AAb, IgETAR-AAb, and IgAT1R-AAb), ANA titers, and the inflammatory cytokine tumor necrosis factor-α (TNF-α) were measured. Multivariable regression models were applied to assess associations between GPCR-AAbs, ANA positivity, and TNF-α levels, adjusting for demographic and clinical covariates, including age. Sex-stratified analyses were performed.

**Results:**

Multiple GPCR-AAbs were significantly associated with ANA positivity, including Igβ1AR-AAb, Igβ2AR-AAb, IgM3MR-AAb, IgM4MR-AAb, IgETAR-AAb, and IgAT1R-AAb. These associations remained robust after adjustment for age and were more pronounced in females. In women, IgM4MR-AAb levels were independently associated with higher TNF-α concentrations (standardized coefficient = 0.28, *p* = 0.004). No significant associations between GPCR-AAbs and TNF-α were observed in men after age adjustment.

**Discussion:**

This large-scale cross-sectional study identifies a selective inflammatory axis linking ANA, TNF-α, and functional GPCR-AAbs—particularly M4 muscarinic receptor autoantibodies—in a sex-specific manner. These findings suggest that GPCR-AAbs may complement ANA as early biomarkers of immune dysregulation and provide novel mechanistic insights into autoimmune activation. GPCR-AAbs may hold clinical relevance for risk stratification and therapeutic targeting in autoimmune diseases.

## 1 Introduction

G protein-coupled receptors (GPCRs) represent the largest family of membrane proteins in humans. They are widely expressed in both non-immune (e.g., neurons, cardiomyocytes, endothelial cells) and immune cells (e.g., monocytes, neutrophils, lymphocytes), where they regulate diverse physiological functions including cell signaling, vascular tone, and immune modulation. Dysregulation of GPCR signaling has been implicated in numerous diseases, ranging from cardiovascular disorders to autoimmune conditions (1, 2). Autoantibodies targeting GPCRs (GPCR-AAbs) can act as functional ligands, mimicking or inhibiting natural ligands and thereby altering receptor-mediated signaling. These antibodies have been detected in various autoimmune diseases and are believed to contribute to disease pathogenesis through mechanisms such as vascular injury, immune cell activation, and tissue remodeling (3–6). Elevated levels of GPCR-AAbs—such as endothelin receptor type A autoantibodies (ETAR-AAb), angiotensin II type 1 receptor autoantibodies (AT1R-AAb), β1/β2-adrenergic receptor autoantibodies (B1AR-AAb, B2AR-AAb), C-X-C chemokine receptor type 3 autoantibodies (CXCR3-Ab) and muscarinic receptor autoantibodies (M3MR-AAb, M4MR-AAb)—have been associated with systemic sclerosis, systemic lupus erythematosus (SLE), lupus nephritis, and Sjögren’s syndrome (6–12). Despite the increasing recognition of GPCR-AAbs, their relationship to classical autoimmune markers—particularly antinuclear antibodies (ANA)—and inflammatory cytokines like tumor necrosis factor-alpha (TNF-α) remains unclear. Prior studies suggest that ANA and GPCR-AAbs may co-occur in autoimmune diseases and could exert synergistic pathogenic effects (13–16). However, direct comparative studies assessing their associations in large cohorts are lacking. In this cross-sectional study, we systematically evaluated serum levels of multiple GPCR-AAbs, ANA titers, TNF-α, IL-6 and CRP in a large cohort of 19,810 individuals. We aimed to elucidate the interplay between functional autoantibodies, classic autoimmune markers, and key cytokines—while also exploring potential sex-specific differences. Understanding these associations may yield novel insights into autoimmune pathophysiology and identify GPCR-AAbs as biomarkers or therapeutic targets.

## 2 Materials and methods

### 2.1 Participants

This cross-sectional study collected data from individuals who underwent testing for G protein-coupled receptor (GPCR) autoantibodies/antibodies at the Institute of Medical Diagnostics (IMD) Berlin-Potsdam, Berlin, Germany, over a five-year period from 2019 to 2024. A total of 19,810 individuals that had been tested for one or multiple GPCR autoantibodies/antibodies were included in this study. These biomarkers comprised β1-adrenergic receptor autoantibodies (B1AR-AAb), β2-adrenergic receptor autoantibodies (B2AR-AAb), M3 muscarinic acetylcholine receptor autoantibody (M3MR-AAb), M4 muscarinic acetylcholine receptor autoantibody (M4MR-AAb), protease-activated receptor-1 antibody (PAR1-Ab), and CXC motif chemokine receptor (CD183) antibody (CXCR3-Ab). Among the 19,810 individuals, 1,937 were additionally tested for antinuclear antibodies (ANA), 4,721 for tumor necrosis factor-alpha (TNF-α), 1,000 for interleukin-6 (IL-6), and 309 for C-reactive protein (CRP).

The GPCR autoantibodies analyzed in this study was introduced into the diagnostic portfolio of the IMD Berlin-Potsdam based on their implicated role in disorders of autonomic regulation and immune dysregulation, particularly post–COVID-19 syndrome and myalgic encephalomyelitis/chronic fatigue syndrome (ME/CFS). Consequently, a substantial proportion of individuals in this cohort were referred for testing in the context of evaluating these conditions. The cohort also includes patients tested by collaborating specialists (e.g., rheumatologists) for suspected classical systemic autoimmune diseases. This real-world cohort therefore represents a mixed clinic-based cohort with symptoms or diagnoses potentially linked to GPCR-mediated pathophysiology.

The Institute of Medical Diagnostics (IMD) Berlin-Potsdam is a certified medical laboratory in accordance with German state regulations for laboratory diagnostics in patient care. All assays performed in this laboratory are subject to rigorous internal and external quality controls, and regular audits are conducted to ensure continued certification for diagnostic testing. This study was conducted in accordance with the ethical standards of the Institute of Medical Diagnostics (IMD) Berlin-Potsdam, which waived the requirement for informed consent. The study adhered to both the institution’s ethical guidelines and the principles outlined in the Declaration of Helsinki regarding the collection and use of data.

### 2.2 Clinical and laboratory parameters

The following clinical and laboratory parameters were collected: sex, age, ANA, anti-double stranded DNA autoantibodies (Anti-dsDNA-AAb), endothelin receptor type A autoantibodies (ETAR-AAb), angiotensin II receptor type 1 autoantibody (AT1R-AAb), β1-adrenergic receptor autoantibodies (B1AR-AAb), β2-adrenergic receptor autoantibodies (B2AR-AAb), M3 muscarinic acetylcholine receptor autoantibody (M3MR-AAb), M4 muscarinic acetylcholine receptor autoantibody (M4MR-AAb), protease-activated receptor 1 antibody (PAR1-Ab), CXC motif chemokine receptor (CD183) antibody (CXCR3-Ab), interleukin-6 (IL-6), C-reactive protein (CRP) and tumor necrosis factor-alpha (TNF-α). All laboratory parameters were analyzed at the Institute of Medical Diagnostics (IMD) Berlin-Potsdam. GPCR autoantibodies were quantified using a quantitative enzyme-linked immunosorbent assay (ELISA) provided by CellTrend GmbH, Germany. IL-6 and TNF-α were quantified by chemiluminescent immunoassay (CLIA) provided by EUROIMMUN, Germany. CRP was measured using high-sensitivity automated immunoturbidimetric assays (Roche Diagnostics, Switzerland).ANA was detected using the gold-standard indirect immunofluorescence test (IIFT) provided by EUROIMMUN, Germany. ANA titers were categorized into four levels: <1:100, 1:100, 1:320, and ≥1:1000.

### 2.3 Statistical analysis

The data were downloaded from the server of the Institute of Medical Diagnostics (IMD) Berlin-Potsdam and organized into an SPSS database. Statistical analyses were conducted using SPSS version 23.0 (IBM Corporation, New York, USA). All parameters were assessed for normality using the Shapiro–Wilk test. Normally distributed parameters are presented as mean ± SEM, whereas non-normally distributed parameters are presented as median (P25, P75). The dataset was divided into two groups based on ANA titers: low ANA (<1:100) and high ANA (≥1:100). For between-group comparisons, the Mann–Whitney U test was applied to skewed (non-normally distributed) variables, whereas the t-test was used for variables that met the assumption of normality. Comparisons of non-normally distributed variables among the three groups were performed using the Kruskal–Wallis test. Continuous variables exhibiting non-normal distributions were subjected to log10 transformation prior to inclusion in subsequent analyses. Correlations between continuous variables were assessed using Pearson’s correlation analysis, whereas correlations involving ordinal variables were analyzed using Spearman’s rank correlation coefficient (Spearman’s rho). Logistic regression analysis was employed for the evaluation of binary variables, whereas linear regression analysis was employed to assess continuous variables. All graphical representations were generated using GraphPad Prism version 10 (GraphPad Software Corporation, California, USA) and are presented as median (P25, P75). Statistical significance was defined as a p-value less than 0.05.

## 3 Results

### 3.1 GPCR autoantibodies are higher in the high ANA group

**Figure 1:**
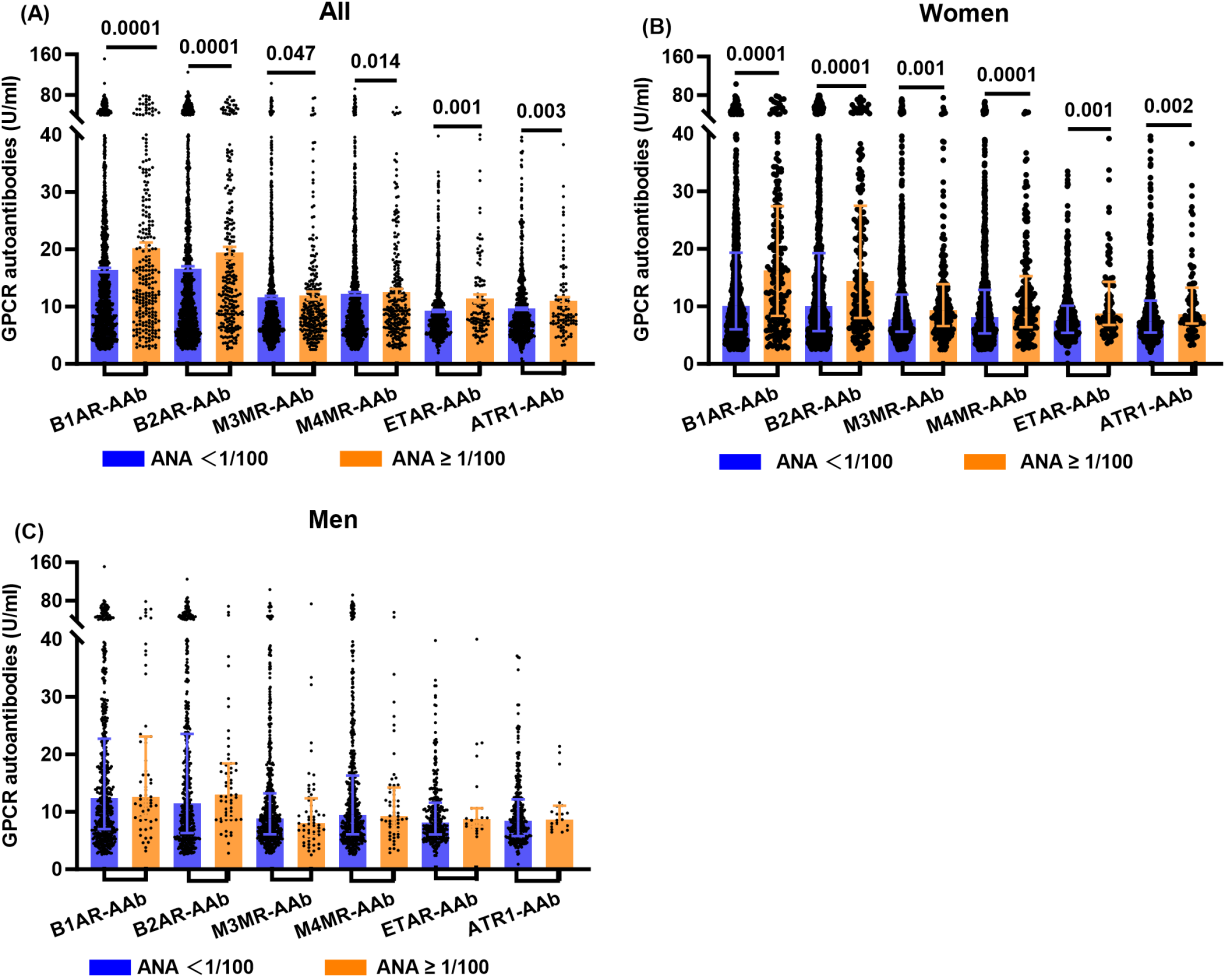
GPCR autoantibody levels across groups: Bar Chart with Scatter Overlay. **(A)** Differences in GPCR autoantibodies B1AR-AAb, B2AR-AAb, M3MR-AAb, M4MR-AAb, ETAR-AAb, and ATR1-AAb between the high and low ANA titer groups in the entire study cohort. **(B)** Differences in GPCR autoantibodies B1AR-AAb, B2AR-AAb, M3MR-AAb, M4MR-AAb, ETAR-AAb, and ATR1-AAb between the high and low ANA titer groups in the female study cohort. **(C)** Differences in GPCR autoantibodies B1AR-AAb, B2AR-AAb, M3MR-AAb, M4MR-AAb, ETAR-AAb, and ATR1-AAb between the high and low ANA titer groups in the male study cohort. Data were shown in median (P25, P75). Between-group comparisons were performed using the Mann–Whitney U test. ANA, Antinuclear antibodies; B1AR-AAb, ß1-Adrenergic receptor autoantibodies; B2AR-AAb, ß2-Adrenergic receptor autoantibodies; M3MR-AAb, M3 muscarinic acetylcholine receptor autoantibody; M4MR-AAb, M4 muscarinic acetylcholine receptor autoantibody; ETAR-AAb, Endothelin receptor type A autoantibodies; ATR1-AAb, Angiotensin II receptor type 1 autoantibody.

Using an ANA titer of 1:100 as the cutoff, the study cohort was divided into two groups: low ANA (<1:100), comprising 1,670 individuals, and high ANA (≥1:100), comprising 267 individuals. The high ANA group was characterized by a higher average age (**Table 1**). Levels of Anti-dsDNA-AAb and several GPCR autoantibodies—including B1AR-AAb, B2AR-AAb, M3MR-AAb, M4MR-AAb, ETAR-AAb, and AT1R-AAb—were significantly elevated in the high ANA group (**Table 1**, **Figure 1A**). No significant differences were observed between the groups in the levels of PAR1-Ab and CXCR3-Ab (**Table 1**). TNF-α and CRP levels were slightly higher in the high-ANA group compared with the low-ANA group; however, these differences were not statistically significant (**Table 1**).

**Table 1:** Comparison of parameters between high and low ANA titer groups.

Parameters	All (N = 1937)		Women (N = 1226)		Men (N = 709)	
	ANA (<1/100)	ANA (≥1/100)	ANA (<1/100)	ANA (≥1/100)	ANA (<1/100)	ANA (≥1/100)
Age	46.14 ± 0.40	49.77 ± 0.95*	45.89 ± 0.49	49.82 ± 1.12*	46.50 ± 0.66	49.60 ± 1.74
Anti-dsDNA-AAb (IU/ml)	16.50 (14.00, 23.75)	28.50 (15.75, 55.00)*	19.50 (14.00, 27.75)	30.00 (17.00, 71.00)*	15.50 (11.00, 22.25)	19.00 (12.50, 29.00)
B1AR-AAb (U/ml)	10.90 (6.40, 20.70)	15.30 (8.70, 26.40)*	10.10 (6.00, 19.35)	16.30 (8.35, 27.40)*	12.40 (6.98, 22.73)	12.60 (9.00, 23.10)
B2AR-AAb (U/ml)	10.60 (6.00, 20.40)	14.15 (8.60, 25.125)*	10.00 (5.70, 19.28)	14.45 (7.975, 27.50)*	11.50 (6.30, 23.55)	12.95 (8.90, 18.40)
M3MR-AAb (U/ml)	8.20 (5.70, 12.53)	9.10 (6.35, 13.50)*	7.70 (5.60, 12.05)	9.35 (6.60, 13.875)*	8.80 (6.10, 13.20)	8.00 (5.80, 12.35)
M4MR-AAb (U/ml)	8.60 (5.60, 14.00)	9.60 (6.60, 14.70)*	8.10 (5.30, 12.90)	9.70 (6.40, 15.25)*	9.50 (6.10, 16.30)	9.25 (6.875, 14.225)
ETAR-AAb (U/ml)	7.65 (5.64, 10.80)	8.73 (6.995, 14.00)*	7.56 (5.41, 10.15)	8.77 (6.7725, 14.275)*	8.14 (6.05, 11.60)	8.73 (7.46, 10.60)
ATR1-AAb (U/ml)	7.74 (5.62, 11.35)	8.67 (7.085, 12.75)*	7.36 (5.45, 11.00)	8.65 (7.04, 13.275)*	8.41 (5.78, 12.15)	8.67 (7.50, 11.05)
PAR1-Ab (U/ml)	3.50 (2.30, 5.90)	3.30 (2.30, 6.95)	3.60 (2.40, 5.90)	3.50 (2.30, 7.65)	3.40 (2.20, 5.80)	2.90 (2.225, 4.925)
CXCR3-Ab (U/ml)	8.70 (6.30, 13.80)	8.60 (6.50, 14.70)	8.30 (6.00, 13.70)	8.35 (6.20, 15.10)	9.50 (6.60, 14.10)	8.85 (7.60, 12.725)
IL6 (pg/ml)	3.40 (2.50, 8.08)	3.20 (2.50, 4.60)	3.80 (2.60, 7.90)	2.80 (2.30, 4.30)	3.20 (2.40, 8.60)	4.50 (2.80, 13.50)
TNF-α (pg/ml)	7.40 (6.00, 9.55)	7.40 (6.10, 10.40)	7.20 (5.80, 9.30)	7.35 (5.825, 10.125)	7.95 (6.40, 9.80)	7.80 (6.50, 10.60)
CRP (mg/l)	2.25 (1.30, 5.20)	2.50 (1.50, 5.35)	2.50 (1.23, 6.75)	2.65 (1.225, 5.725)	2.20 (1.50, 4.70)	2.30 (1.80, 34.30)

All parameters were assessed for normality using the Shapiro–Wilk test. Normally distributed parameters are presented as mean ± SEM, whereas non-normally distributed parameters are presented as median (P25, P75). For between-group comparisons, the Mann–Whitney U test was applied to skewed (non-normally distributed) variables, whereas the t-test was used for variables that met the assumption of normality. * p < 0.05, statistically significant. ANA, Antinuclear antibodies; Anti-dsDNA-AAb, Anti-double stranded DNA autoantibodies; B1AR-AAb, ß1-Adrenergic receptor autoantibodies; B2AR-AAb, ß2-Adrenergic receptor autoantibodies; M3MR-AAb, M3 muscarinic acetylcholine receptor autoantibody; M4MR-AAb, M4 muscarinic acetylcholine receptor autoantibody; ETAR-AAb, Endothelin receptor type A autoantibodies; ATR1-AAb, Angiotensin II receptor type 1 autoantibody; PAR1-Ab, Protease-activated Receptor 1 antibody; CXCR3-Ab, CXC motif chemokine receptors antibody (CD183); IL6, Interleukin 6; TNF-α, Tumor Necrosis Factor alpha; CRP, C-reactive protein.

### 3.2 Sex-specific differences in GPCR autoantibodies between low and high ANA groups

The study cohort was stratified by sex, and each subgroup was further divided based on ANA titers using the 1:100 threshold. The analysis revealed that the levels of GPCR autoantibodies—including B1AR-AAb, B2AR-AAb, M3MR-AAb, M4MR-AAb, ETAR-AAb, and AT1R-AAb—remained significantly different between the high and low ANA groups within the female cohort. However, no such differences were observed in the male cohort (**Table 1**, **Figures 1B, C**).

### 3.3 ANA is positively associated with the GPCR autoantibodies lgB1AR-AAb, lgB2AR-AAb, lgM3MR-AAb, lgM4MR-AAb, lgETAR-AAb, and lgAT1R-AAb

Correlation analysis demonstrated positive associations between ANA titers and age, as well as with GPCR autoantibodies including lgB1AR-AAb, lgB2AR-AAb, lgM3MR-AAb, lgM4MR-AAb, lgETAR-AAb, and lgAT1R-AAb (**Table 2**). Logistic regression analysis, adjusted for age to control for potential confounding, identified lgB1AR-AAb (OR = 2.22, p = 0.0001), lgB2AR-AAb (OR = 1.98, p = 0.0001), lgETAR-AAb (OR = 4.99, p = 0.001), and lgAT1R-AAb (OR = 3.27, p = 0.007) as independent positive predictors of elevated ANA titers (**Table 3**). These associations also displayed sex-specific patterns. In the female cohort, both correlation and regression analyses confirmed positive relationships between ANA and lgB1AR-AAb, lgB2AR-AAb, lgM3MR-AAb, lgM4MR-AAb, lgETAR-AAb, and lgAT1R-AAb. In contrast, these associations were not observed in the male cohort (**Tables 2**, **3**). Notably, after age adjustment, ANA remained associated with IgM3MR-AAb and IgM4MR-AAb in females, but not in the entire cohort.

**Table 2:** Correlation analysis between ANA and other parameters.

Parameters	ANA (All)			ANA (Women)			ANA (Men)		
	R	P	N	R	P	N	R	P	N
Age	0.07	0.001	1937	0.09	0.002	1226	0.05	0.229	709
lgB1AR-AAb	0.12	0.0001	1765	0.16	0.0001	1114	0.04	0.283	649
lgB2AR-AAb	0.11	0.0001	1705	0.14	0.0001	1074	0.05	0.250	629
lgM3MR-AAb	0.05	0.041	1751	0.10	0.001	1113	-0.05	0.252	636
lgM4MR-AAb	0.06	0.011	1751	0.11	0.0001	1110	-0.01	0.832	639
lgETAR-AAb	0.11	0.001	875	0.14	0.001	559	0.07	0.208	315
lgATR1-AAb	0.10	0.002	878	0.13	0.002	559	0.06	0.310	318
lgPAR1-Ab	0.02	0.613	611	0.04	0.497	379	-0.03	0.668	231
lgCXCR3-Ab	0.01	0.776	680	0.02	0.688	428	0.00	0.966	251
lgIL6	-0.04	0.561	216	-0.11	0.253	110	0.09	0.385	106
lgTNF-α	0.00	0.944	929	0.02	0.668	562	0.03	0.614	366
lgCRP	0.05	0.560	117	0.02	0.874	75	0.07	0.653	41

lg = log base 10. A Spearman’s ‘rho’ Correlation was applied for the correlation analysis. ANA grades: < 1:100; 1:100; 1:320; ≥1:1000. ANA, Antinuclear antibodies; Anti-dsDNA-AAb, Anti-double stranded DNA autoantibodies; ETAR-AAb, Endothelin receptor type A autoantibodies; ATR1-AAb, Angiotensin II receptor type 1 autoantibody; B1AR-AAb, ß1-Adrenergic receptor autoantibodies; B2AR-AAb, ß2-Adrenergic receptor autoantibodies; M3MR-AAb, M3 muscarinic acetylcholine receptor autoantibody; M4MR-AAb, M4 muscarinic acetylcholine receptor autoantibody; PAR1-Ab, Protease-activated Receptor 1 antibody; CXCR3-Ab, CXC motif chemokine receptors antibody (CD183); IL6, Interleukin 6; TNF-α, Tumor Necrosis Factor alpha; CRP, C-reactive protein. Red indicates significant p values.

**Table 3:** The logistic regression analysis of ANA as a dependent variable.

Independent variable	Dependent variable: ANA					
	All		Women		Men	
	OR	p	OR	p	OR	p
lgB1AR-AAb	2.22	0.0001	2.90	0.0001	1.37	0.433
lgB2AR-AAb	1.98	0.0001	2.50	0.0001	1.23	0.599
lgM3MR-AAb	1.22	0.420	1.85	0.028	0.41	0.101
lgM4MR-AAb	1.35	0.190	2.11	0.007	0.69	0.432
lgETAR-AAb	4.99	0.001	6.63	0.001	3.81	0.167
lgATR1-AAb	3.27	0.007	3.79	0.006	2.57	0.345

lg = log base 10. lgETAR-AAb, lgATR1-AAb, lgB1AR-AAb, lgB2AR-AAb, lgM3MR-AAb, and lgM4MR-AAb were modelled with age respectively. OR: Odds ratio; ANA was divided into two groups: ANA (<1:100) and ANA (≥1:100). ANA, Antinuclear antibodies; B1AR-AAb, ß1-Adrenergic receptor autoantibodies; B2AR-AAb, ß2-Adrenergic receptor autoantibodies; M3MR-AAb, M3 muscarinic acetylcholine receptor autoantibody; M4MR-AAb, M4 muscarinic acetylcholine receptor autoantibody; ETAR-AAb, Endothelin receptor type A autoantibodies; ATR1-AAb, Angiotensin II receptor type 1 autoantibody. Red indicates significant p values.

### 3.4 The GPCR autoantibodies lgM4MR-AAb are positively correlated with lgTNF-α

**Figure 2:**
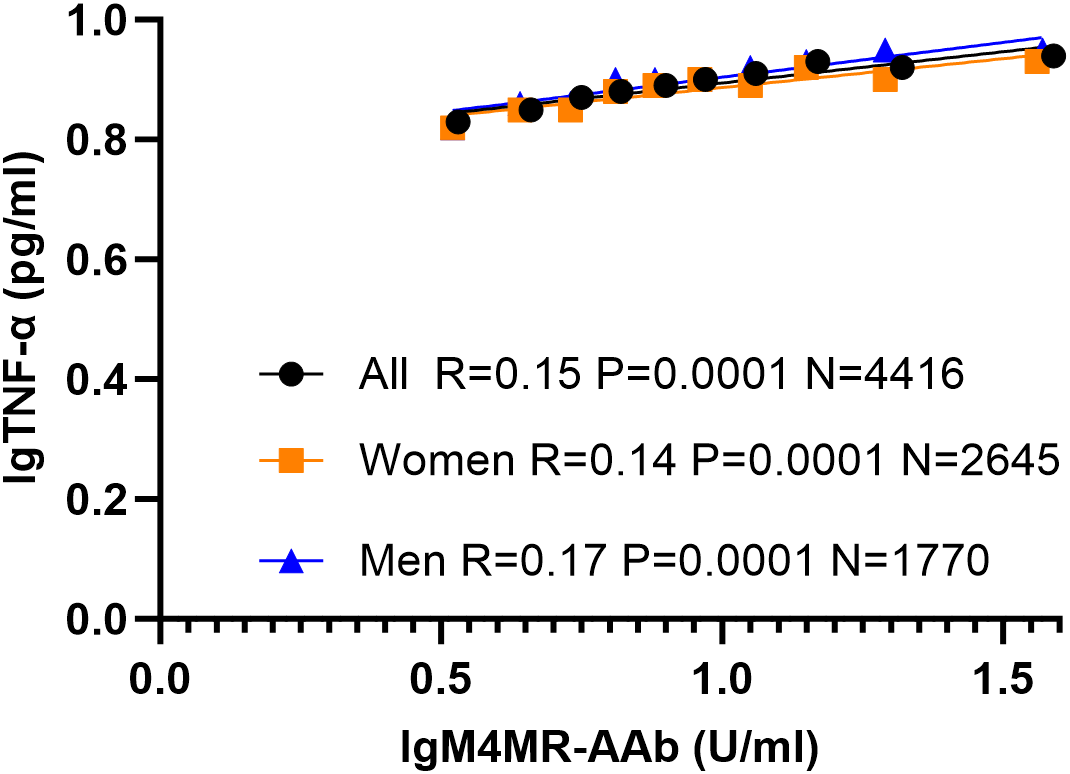
Correlation between lgTNF-α and lgM4MR-AAb in different study cohorts. The study cohort was divided into 10 groups based on the percentile of the actual lgM4MR-AAb value (every 10%), and the mean and SEM of lgTNF-α and lgM4MR-AAb in each group were plotted. Pearson’s correlation was applied for the correlation analysis. M4MR-AAb, M4 muscarinic acetylcholine receptor autoantibody; TNF-α, Tumor Necrosis Factor alpha. lg = log base 10.

Correlation analyses showed that lgTNF-α levels were positively associated with several GPCR autoantibodies—including lgB1AR-AAb, lgB2AR-AAb, lgM3MR-AAb, and lgM4MR-AAb—in both the female and male cohorts (**Table 4**). Furthermore, linear regression analysis incorporating age and all GPCR autoantibodies—lgB1AR-AAb, lgB2AR-AAb, lgM3MR-AAb, lgM4MR-AAb, lgETAR-AAb, lgAT1R-AAb, lgPAR1-Ab, and lgCXCR3-Ab—identified only lgM4MR-AAb as an independent predictor of elevated lgTNF-α (**Table 5**) in both the overall cohort (Standardized Coefficient = 0.24, p = 0.002) and the female cohort (Standardized Coefficient = 0.28, p = 0.004), but not in the male cohort. **Figure 2** illustrates the correlation between lgM4MR-AAb and lgTNF-α. lgCXCR3-Ab (Standardized Coefficient = 0.08, p = 0.043) showed a weak association with lgTNF-α only in the overall study population. The relationship between lgB2AR-AAb and lgTNF-α showed inconsistent directions in the regression model and correlation analysis, and the association was very weak.

**Table 4:** Correlation of lgTNF-α with other parameters.

Parameters	lgTNF-α								
	All			Women			Men		
	R	P	N	R	P	N	R	P	N
Age	-0.05	0.0001	4721	-0.05	0.004	2840	-0.06	0.016	1880
lgB1AR-AAb	0.09	0.0001	4352	0.08	0.0001	2601	0.10	0.0001	1750
lgB2AR-AAb	0.06	0.0001	4229	0.05	0.015	2515	0.08	0.001	1713
lgM3MR-AAb	0.11	0.0001	4413	0.09	0.0001	2649	0.14	0.0001	1763
lgM4MR-AAb	0.15	0.0001	4416	0.14	0.0001	2645	0.17	0.0001	1770
lgETAR-AAb	0.03	0.292	1375	-0.02	0.614	807	0.09	0.038	567
lgATR1-AAb	-0.01	0.697	1369	-0.05	0.191	810	0.04	0.362	558
lgPAR1-Ab	0.01	0.692	958	0.01	0.790	553	0.02	0.664	404
lgCXCR3-Ab	0.07	0.025	1065	0.06	0.134	623	0.08	0.112	441

lg = log base 10. Pearson’s correlation was applied for the correlation analysis. B1AR-AAb, ß1-Adrenergic receptor autoantibodies; B2AR-AAb, ß2-Adrenergic receptor autoantibodies; M3MR-AAb, M3 muscarinic acetylcholine receptor autoantibody; M4MR-AAb, M4 muscarinic acetylcholine receptor autoantibody; ETAR-AAb, Endothelin receptor type A autoantibodies; ATR1-AAb, Angiotensin II receptor type 1 autoantibody; PAR1-Ab, Protease-activated Receptor 1 antibody; CXCR3-Ab, CXC motif chemokine receptors antibody (CD183); TNF-α, Tumor Necrosis Factor alpha. Red indicates significant p values.

**Table 5:** The linear regression analysis of lgTNF-α as a dependent variable.

Independent variable	Dependent variable: lgTNF-α					
	All		Women		Men	
	Standardized coefficients	p	Standardized coefficients	p	Standardized coefficients	p
lgB1AR-AAb	0.15	0.060	0.18	0.091	0.12	0.314
lgB2AR-AAb	-0.18	0.018	-0.22	0.023	-0.07	0.555
lgM3MR-AAb	-0.10	0.250	-0.14	0.230	-0.06	0.661
lgM4MR-AAb	0.24	0.002	0.28	0.004	0.15	0.235
lgETAR-AAb	0.09	0.344	0.01	0.949	0.19	0.224
lgATR1-AAb	-0.14	0.110	-0.07	0.571	-0.26	0.075
lgPAR1-Ab	0.00	0.914	0.00	0.982	-0.02	0.770
lgCXCR3-Ab	0.08	0.043	0.10	0.072	0.07	0.257

lg = log base 10. Regression model: age, lgB1AR-AAb, lgB2AR-AAb, lgM3MR-AAb, lgM4MR-AAb, lgETAR-AAb, lgATR1-AAb, lgPAR1-Ab, and lgCXCR3-Ab. B1AR-AAb, ß1-Adrenergic receptor autoantibodies; B2AR-AAb, ß2-Adrenergic receptor autoantibodies; M3MR-AAb, M3 muscarinic acetylcholine receptor autoantibody; M4MR-AAb, M4 muscarinic acetylcholine receptor autoantibody; ETAR-AAb, Endothelin receptor type A autoantibodies; ATR1-AAb, Angiotensin II receptor type 1 autoantibody; PAR1-Ab, Protease-activated Receptor 1 antibody; CXCR3-Ab, CXC motif chemokine receptors antibody (CD183); TNF-α, Tumor Necrosis Factor alpha. Red indicates significant p values.

In addition, the associations between GPCR autoantibodies and the inflammatory markers lgIL6 and lgCRP were also examined (**Supplementary Tables S1**, **S2**). The correlations between lgIL6 and lgB1AR-AAb or lgM4MR-AAb disappeared after adjusting for age (**Supplementary Table S3**). lgCRP was not associated with any GPCR autoantibody except for age; however, it should be noted that the sample size for lgCRP was limited (n = 309).

## 4 Discussion

### 4.1 Principal findings

In this large clinic-based cohort, we observed that elevated ANA titers were significantly associated with higher levels of multiple GPCR-AAbs, including lgB1AR-AAb, lgB2AR-AAb, lgM3MR-AAb, lgM4MR-AAb, lgETAR-AAb, and lgATR1-AAb, particularly in women. Among these autoantibodies, M4MR-AAb demonstrated strong correlation with serum TNF-α concentrations. These findings suggest a previously underappreciated link between classical autoimmunity markers and functionally active autoantibodies with pro-inflammatory potential.

### 4.2 Cooperative interaction of ANA and GPCR autoantibodies

In autoimmune diseases, both T and B lymphocytes are excessively activated (6). Activated B cells differentiate into plasma cells, producing a broad spectrum of autoantibodies, with antinuclear antibodies (ANAs) and GPCR autoantibodies being particularly prominent (17). Fluctuations in ANA levels are often correlated with disease activity. According to the American College of Rheumatology (ACR), ANA titers measured by indirect immunofluorescence (IIF) are considered the gold standard for the diagnosis of SLE (18, 19). GPCR autoantibodies exert biological effects by binding to their corresponding receptors, mimicking the actions of endogenous ligands. Therefore, GPCR autoantibodies are regarded as effector molecules in specific pathological mechanisms of autoimmune diseases, such as lupus myocarditis (2, 12) and microvascular dysfunction in SSc (20, 21). Although both GPCR autoantibodies and ANAs are frequently detected in autoimmune diseases, the relationship between them remains unclear. In the present study, we investigated the association between ANA titers and GPCR autoantibodies by stratifying participants according to ANA levels. Our findings demonstrated that individuals with higher ANA titers exhibited elevated levels of specific GPCR autoantibodies. This association was more pronounced in the female cohort, consistent with the higher prevalence of autoimmune diseases in women. Our findings support the hypothesis that ANA and GPCR autoantibodies not only coexist but may also exhibit functional interactions.

Among the various GPCR autoantibodies examined, those targeting receptors involved in the regulation of the cardiovascular and autonomic nervous systems—namely ETAR-AAb, ATR1-AAb, B1AR-AAb, B2AR-AAb, M3MR-AAb, and M4MR-AAb—showed the strong correlations with ANA titers. ETAR-AAb and AT1R-AAb can activate a variety of intracellular signaling pathways in both non-immune cells (such as endothelial and smooth muscle cells) and immune cells through binding to their respective receptors, ETAR and AT1R (21–24). A study involving 478 patients with SSc revealed that ETAR-AAb and AT1R-AAb levels were elevated, and higher levels of these autoantibodies were associated with more severe clinical symptoms (21). Another study further demonstrated that, compared to other forms of pulmonary arterial hypertension (PAH), patients with SSc-associated PAH exhibited significantly higher and more prevalent levels of ETAR-AAb and AT1R-AAb (20). B1AR-AAb has been shown to induce calcium overload in cardiomyocytes and promote the release of inflammatory cytokines through excessive stimulation of B1AR (25–27). This may represent a key pathological mechanism underlying SSc-related cardiac involvement and lupus myocarditis (2, 12). In autoimmune diseases—particularly SLE and SSc—B2AR-AAb contributes to inflammation, disruption of immune tolerance, and microvascular dysfunction (28, 29). M3MR-AAb is highly prevalent among patients with systemic sclerosis (SSc) who exhibit gastrointestinal involvement. Emerging evidence indicates that M3MR-AAb disrupts cholinergic neurotransmission, thereby mechanistically contributing to the gastrointestinal dysmotility characteristic of SSc (6, 30, 31). In Sjögren’s syndrome, M3MR-AAb blocks parasympathetic neurotransmission, leading to dry eyes/mouth, gastrointestinal dysmotility, and bladder dysfunction (32). Similar to M3MR-AAb, M4MR-AAb appears to mediate gastrointestinal dysfunction in autoimmune diseases (33) and may also contribute to autonomic nervous system (ANS) dysregulation by disrupting cholinergic modulation of cardiovascular function (34). Therefore, autoimmune diseases characterized predominantly by microvascular injury, myocarditis, or autonomic nervous system dysfunction, monitoring GPCR autoantibodies—especially ETAR-AAb, ATR1-AAb, B1AR-AAb, B2AR-AAb, M3MR-AAb, and M4MR-AAb—may offer a more sensitive indicator of disease activity than conventional autoantibodies. Further investigation into the cooperative relationship between ANAs and GPCR autoantibodies may facilitate the development of targeted therapeutic strategies for organ-specific damage in autoimmune diseases.

### 4.3 Association of GPCR autoantibodies with inflammatory cytokines

Excessive activation of immune cells and the overproduction of inflammatory cytokines are key pathological mechanisms in autoimmune diseases. Inflammatory cytokine TNF-α plays central role in the immune response and is involved in the pathogenesis of various autoimmune diseases (35). In this study cohort, we observed a positive correlation between M4MR-AAb levels and TNF-α. Mechanistically, M4 receptors are expressed on certain immune cell populations (e.g., T and B lymphocytes), forming part of the cholinergic regulatory machinery of the immune system (36). Under physiological conditions, acetylcholine engagement of these receptors modulates cytokine release—typically exerting an inhibitory effect on pro-inflammatory pathways. Animal studies have demonstrated that stimulation of central muscarinic receptors suppresses peripheral TNF levels during endotoxemia (37), and muscarinic signaling within the spleen can alter cytokine expression profiles of immune cells (38). Thus, blockade of M4 receptor function on immune cells by autoantibodies could impair this anti-inflammatory regulatory mechanism. Our findings are consistent with this theoretical framework. Although the regulatory mechanisms linking M4MR-AAb to TNF-α have not yet been fully elucidated, multiple studies have reported concomitant elevations of M4MR-AAb and TNF-α, as well as T- and B-cell activation, in several disease states (39, 40). Notably, in patients with myalgic encephalomyelitis/chronic fatigue syndrome (ME/CFS) undergoing B-cell–depleting therapy with rituximab, clinical improvement was accompanied by a decline in elevated M4MR-AAb levels (40). Collectively, existing evidence together with our current analysis suggests that M4MR-AAbs and TNF-α constitute a critical nexus between autoimmunity and inflammation, and that their interaction may contribute to sustained immune activation and inflammatory manifestations in certain autoimmune disorders.

### 4.4 Sex-specific immune modulation

The observed female predominance in GPCR-AAb–TNF-α correlations aligns with well-established sex differences in immune regulation. Estrogen is known to amplify B cell activity and antibody production while also modulating T cell differentiation (41, 42). These sex-specific effects may explain the greater vulnerability of women to both ANA formation and GPCR-AAb–mediated inflammation.

### 4.5 Clinical implications

This study is based on a clinic-based cohort of 19,810 individuals, in which we observed statistically significant but weak associations between ANA and GPCR autoantibodies. GPCR autoantibodies may be broadly present in the general population, and elevated levels may indicate increased susceptibility to autoimmune diseases. GPCR-AAbs may serve as biomarkers of low-grade inflammation and immune activation in ANA-positive individuals, even in the absence of overt autoimmune disease. Their association with TNF-α suggests a role in the pathophysiological transition from preclinical to clinical autoimmunity. These findings raise the possibility of using GPCR-AAb profiles for early risk stratification and personalized monitoring strategies—particularly in women.

### 4.6 GPCR autoantibodies in post-COVID syndrome and chronic fatigue – links to our findings

Elevated GPCR autoantibodies, particularly against β-adrenergic and muscarinic receptors, are found in individuals with post-COVID syndrome and chronic fatigue (ME/CFS) and are thought to drive symptoms like fatigue, cognitive impairment, and autonomic dysfunction (43, 44). Given that 88.43% (n = 17,517) of the data were collected between 2022 and 2024, the dataset primarily represents the post–COVID-19 period (**Supplementary Figure S1**). Our findings suggest that elevated GPCR autoantibodies in post–COVID-19 individuals may actively contribute to immune dysregulation via effects on TNF-α, thereby playing a potential role in persistent post–COVID-19 syndrome. Although we compared autoantibody levels across the three testing-year groups (2019, 2020–2021, 2022–2024) using the Kruskal–Wallis test, no significant differences were detected (**Supplementary Table S4**). The pronounced imbalance in sample size across the groups may have reduced the statistical power of these comparisons.

## 5 Limitations

This study has several limitations. First, its cross-sectional design precludes causal inference. Longitudinal follow-up is needed to determine whether elevated GPCR-AAbs and TNF-α levels precede or result from immune activation. Second, the large clinic-based cohort consisted primarily of older individuals, potentially limiting generalizability to younger cohorts. Third, given that the data were obtained from a diagnostic laboratory, corresponding clinical symptom information was unavailable; additionally, only a limited set of inflammatory markers (IL-6, TNF-α, and CRP) was measured. Consequently, we are currently unable to establish a systematic and comprehensive link between mechanistic findings and clinical phenotypes, thereby limiting our ability to provide a more in-depth interpretation of the biological significance of these GPCR autoantibodies. Future longitudinal or mechanistic studies will be essential to determine whether these autoantibodies have predictive or pathogenic relevance. Lastly, the smaller number of male participants may have resulted in insufficient statistical power to detect weaker associations in men.

## 6 Conclusions

This is the first large-scale study to systematically demonstrate that elevated ANA titers are associated with increased levels of functional GPCR-AAbs, which in turn correlate with TNF-α in a sex-specific manner. These results provide new insights into the interface between classical autoimmune markers and functional autoantibodies and suggest a novel inflammatory axis potentially relevant to disease prediction and early intervention. Future research should focus on longitudinal validation, functional mechanistic studies, and integration of GPCR-AAbs into biomarker panels for autoimmune risk prediction.

## Data Availability

The raw data supporting the conclusions of this article will be made available by the authors, without undue reservation.
