# Supplementary material for: Associations between functional autoantibodies targeting GPCRs, antinuclear antibodies, and inflammatory cytokines TNF-α: a cross-sectional study of 19,810 individuals
Source: Front Immunol. 2026 Jan 19;16:1743537. doi: 10.3389/fimmu.2025.1743537 (PMC12861881; doi:10.3389/fimmu.2025.1743537)
Supplement: Supplementary file 1 [file Table1.docx]

Supplementary Material

# Supplementary Tables

**Table S1.** Correlation of lgIL6 with other parameters.

| **Parameters** | **lgIL6** | | | | | | | | |
| --- | --- | --- | --- | --- | --- | --- | --- | --- | --- |
|  | **All** | | | **Women** | | | **Men** | | |
|  | **R** | **P** | **N** | **R** | **P** | **N** | **R** | **P** | **N** |
| Age | 0.03 | 0.327 | 1000 | 0.05 | 0.194 | 596 | 0.01 | 0.912 | 404 |
| lgB1AR-AAb | 0.07 | 0.044 | 895 | 0.09 | 0.047 | 523 | 0.04 | 0.479 | 372 |
| lgB2AR-AAb | 0.03 | 0.319 | 891 | 0.05 | 0.251 | 522 | 0.01 | 0.884 | 369 |
| lgM3MR-AAb | 0.06 | 0.094 | 919 | 0.05 | 0.275 | 545 | 0.07 | 0.195 | 374 |
| lgM4MR-AAb | 0.07 | 0.049 | 919 | 0.06 | 0.137 | 537 | 0.06 | 0.212 | 382 |
| lgETAR-AAb | 0.08 | 0.116 | 371 | 0.10 | 0.139 | 226 | 0.04 | 0.633 | 145 |
| lgATR1-AAb | 0.04 | 0.448 | 359 | 0.06 | 0.403 | 218 | -0.01 | 0.893 | 141 |
| lgPAR1-Ab | -0.04 | 0.534 | 267 | -0.02 | 0.799 | 158 | -0.07 | 0.493 | 109 |
| lgCXCR3-Ab | 0.01 | 0.818 | 281 | 0.02 | 0.790 | 166 | -0.01 | 0.890 | 115 |

**Notes:** lg = log base 10. Pearson's correlation was applied for the correlation analysis. B1AR-AAb:ß1-Adrenergic receptor autoantibodies; B2AR-AAb: ß2-Adrenergic receptor autoantibodies; M3MR-AAb: M3 muscarinic acetylcholine receptor autoantibody; M4MR-AAb: M4 muscarinic acetylcholine receptor autoantibody; ETAR-AAb: Endothelin receptor type A autoantibodies; ATR1-AAb: Angiotensin II receptor type 1 autoantibody; PAR1-Ab: Protease-activated Receptor 1 antibody; CXCR3-Ab: CXC motif chemokine receptors antibody (CD183); IL6: Interleukin 6.

**Table S2.** Correlation of lgCRP with other parameters.

| **Parameters** | **lgCRP** | | | | | | | | |
| --- | --- | --- | --- | --- | --- | --- | --- | --- | --- |
|  | **All** | | | **Women** | | | **Men** | | |
|  | **R** | **P** | **N** | **R** | **P** | **N** | **R** | **P** | **N** |
| Age | -0.13 | 0.019 | 309 | -0.18 | 0.013 | 197 | -0.07 | 0.457 | 111 |
| lgB1AR-AAb | -0.01 | 0.851 | 284 | -0.05 | 0.478 | 181 | -0.05 | 0.478 | 181 |
| lgB2AR-AAb | 0.03 | 0.673 | 276 | 0.0001 | 0.997 | 171 | 0.000 | 0.997 | 171 |
| lgM3MR-AAb | -0.05 | 0.420 | 286 | -0.03 | 0.732 | 184 | -0.03 | 0.732 | 184 |
| lgM4MR-AAb | -0.04 | 0.509 | 290 | -0.08 | 0.303 | 184 | -0.08 | 0.303 | 184 |
| lgETAR-AAb | -0.03 | 0.775 | 121 | 0.07 | 0.571 | 76 | 0.07 | 0.571 | 76 |
| lgATR1-AAb | 0.003 | 0.975 | 118 | 0.12 | 0.301 | 74 | 0.12 | 0.301 | 74 |
| lgPAR1-Ab | -0.09 | 0.387 | 96 | -0.01 | 0.928 | 59 | -0.01 | 0.928 | 59 |
| lgCXCR3-Ab | 0.07 | 0.520 | 99 | 0.03 | 0.802 | 65 | 0.03 | 0.802 | 65 |

**Notes:** lg = log base 10. Pearson's correlation was applied for the correlation analysis. B1AR-AAb:ß1-Adrenergic receptor autoantibodies; B2AR-AAb: ß2-Adrenergic receptor autoantibodies; M3MR-AAb: M3 muscarinic acetylcholine receptor autoantibody; M4MR-AAb: M4 muscarinic acetylcholine receptor autoantibody; ETAR-AAb: Endothelin receptor type A autoantibodies; ATR1-AAb: Angiotensin II receptor type 1 autoantibody; PAR1-Ab: Protease-activated Receptor 1 antibody; CXCR3-Ab: CXC motif chemokine receptors antibody (CD183); CRP: C-reactive protein.

**Table S3.** The linear regression analysis of lgIL6 as a dependent variable.

| **Independent Variable** | **Dependent Variable: lgIL6** | | | | | |
| --- | --- | --- | --- | --- | --- | --- |
|  | **All** | | **Women** | | **Men** | |
|  | **Standardized Coefficients** | **p** | **Standardized Coefficients** | **p** | **Standardized Coefficients** | **p** |
| lgB1AR-AAb | 0.07 | 0.307 | 0.03 | 0.901 | 0.25 | 0.233 |
| lgB2AR-AAb | 0.09 | 0.527 | -0.06 | 0.755 | -0.62 | 0.020 |
| lgM3MR-AAb | -0.27 | 0.083 | 0.07 | 0.761 | 0.43 | 0.086 |
| lgM4MR-AAb | 0.20 | 0.201 | 0.19 | 0.359 | 0.09 | 0.716 |
| lgETAR-AAb | 0.18 | 0.245 | 0.14 | 0.376 | 0.25 | 0.278 |
| lgATR1-AAb | 0.17 | 0.182 | -0.11 | 0.580 | -0.38 | 0.086 |
| lgPAR1-Ab | -0.22 | 0.115 | -0.02 | 0.854 | 0.002 | 0.984 |
| lgCXCR3-Ab | -0.01 | 0.883 | -0.06 | 0.650 | -0.07 | 0.543 |

**Notes:** lg = log base 10. Regression model: age, lgB1AR-AAb, lgB2AR-AAb, lgM3MR-AAb, lgM4MR-AAb, lgETAR-AAb, lgATR1-AAb, lgPAR1-Ab, and lgCXCR3-Ab. B1AR-AAb:ß1-Adrenergic receptor autoantibodies; B2AR-AAb: ß2-Adrenergic receptor autoantibodies; M3MR-AAb: M3 muscarinic acetylcholine receptor autoantibody; M4MR-AAb: M4 muscarinic acetylcholine receptor autoantibody; ETAR-AAb: Endothelin receptor type A autoantibodies; ATR1-AAb: Angiotensin II receptor type 1 autoantibody; PAR1-Ab:Protease-activated Receptor 1 antibody; CXCR3-Ab: CXC motif chemokine receptors antibody (CD183); IL6: Interleukin 6.

**Table S4.** Intergroup comparison of parameters across years.

| **Parameters** | **Year of testing**  **2019** | **Year of testing**  **2020-2021** | **Year of testing**  **2022-2024** |
| --- | --- | --- | --- |
| **B1AR-AAb (U/ml)** | 12.05 (6.90, 20.25) | 11.50 (6.70, 22.30) | 11.60 (6.80, 21.60) |
| **B2AR-AAb (U/ml)** | 11.40 (6.10, 21.33) | 10.90 (6.30, 22.50) | 11.20 (6.30, 22.30) |
| **M3MR-AAb (U/ml)** | 8.40 (5.80, 12.70) | 8.20 (5.60, 13.50) | 8.30 (5.70, 13.30) |
| **M4MR-AAb (U/ml)** | 8.70 (5.50, 14.80) | 8.60 (5.40, 15.00) | 8.70 (5.60, 14.80) |
| **ETAR-AAb (U/ml)** | 7.53 (6.02, 10.70) | 7.84 (5.88, 12.00) | 7.93 (5.96, 11.30) |
| **ATR1-AAb (U/ml)** | 7.72 (5.79, 11.90) | 7.97 (5.88, 11.95) | 8.08 (5.88, 11.80) |
| **PAR1-Ab (U/ml)** | 3.00 (1.90, 4.50) | 3.50 (2.40, 5.20) | 3.40 (2.20, 5.70) |
| **CXCR3-Ab (U/ml)** | 9.00 (6.40, 12.85) | 8.50 (6.30, 13.30) | 8.70 (6.30, 13.20) |
| **IL6 (pg/ml)** | 4.70 (2.60, 7.70) | 3.60 (2.50, 7.95) | 3.30 (2.50, 5.98) |
| **TNF-α (pg/ml)** | 7.35 (5.45, 9.80) | 7.10 (5.70, 9.10) | 7.30 (5.80, 9.50) |
| **CRP (mg/l)** | - | 2.40 (1.60, 4.90) | 2.00 (1.30, 4.20) |

Note: All parameters were assessed for normality using the Shapiro–Wilk test. Non-normally distributed parameters are presented as median (P25, P75). Between-group comparisons were performed using the Kruskal–Wallis test, and no statistically significant differences in autoantibody levels were observed among the three groups. B1AR-AAb: ß1-Adrenergic receptor autoantibodies; B2AR-AAb: ß2-Adrenergic receptor autoantibodies; M3MR-AAb: M3 muscarinic acetylcholine receptor autoantibody; M4MR-AAb: M4 muscarinic acetylcholine receptor autoantibody; ETAR-AAb: Endothelin receptor type A autoantibodies; ATR1-AAb: Angiotensin II receptor type 1 autoantibody; PAR1-Ab:Protease-activated Receptor 1 antibody; CXCR3-Ab: CXC motif chemokine receptors antibody (CD183); IL6: Interleukin 6; TNF-α: Tumour Necrosis Factor alpha; CRP: C-reactive protein.

# Supplementary Figure


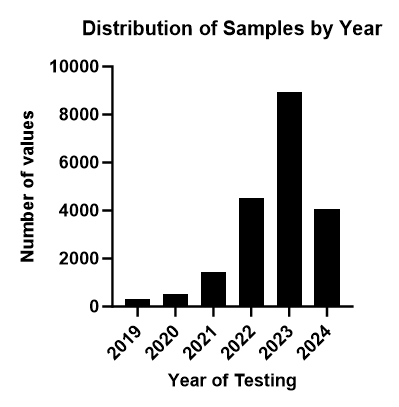


**Figure S1.** Distribution of samples by year. A total of 88.43% (n = 17,517) of the samples were collected between 2022 and 2024, 9.91% (n = 1,963) between 2020 and 2021, and 1.67% (n = 330) in 2019.
